# Renal Senescence, Telomere Shortening and Nitrosative Stress in Feline Chronic Kidney Disease

**DOI:** 10.3390/vetsci8120314

**Published:** 2021-12-08

**Authors:** Jessica Quimby, Andrea Erickson, Shannon Mcleland, Rachel Cianciolo, David Maranon, Katharine Lunn, Jonathan Elliott, Jack Lawson, Ann Hess, Rene Paschall, Susan Bailey

**Affiliations:** 1 Department of Veterinary Clinical Sciences, The Ohio State University, Columbus, OH 43210, USA; Erickson.293@osu.edu (A.E.); Paschall.7@osu.edu (R.P.); 2 International Veterinary Renal Pathology Service, Department of Veterinary Biosciences, The Ohio State University, Columbus, OH 43210, USA; smcleland@hotmail.com (S.M.); Cianciolo.14@osu.edu (R.C.); 3 Department of Environmental and Radiological Health Sciences, Colorado State University, Fort Collins, CO 80521-1618, USA; David.Maranon@colostate.edu (D.M.); Susan.Bailey@colostate.edu (S.B.); 4 Department of Clinical Sciences, North Carolina State University, Raleigh, NC 27695-0001, USA; kflunn@ncsu.edu; 5 Department of Comparative Biomedical Sciences, Royal Veterinary College, London E16 2PX, UK; jelliott@rvc.ac.uk; 6 Department of Clinical Sciences and Services, Royal Veterinary College, Herts AL9 7TA, UK; jlawson@rvc.ac.uk; 7 Department of Statistics, Colorado State University, Fort Collins, CO 80521-4593, USA; Ann.Hess@colostate.edu

**Keywords:** feline, cat, renal disease, senescence, aging

## Abstract

Kidney tissues from cats with naturally occurring chronic kidney disease (CKD) and adult and senior cats without CKD were assessed to determine whether telomere shortening and nitrosative stress are associated with senescence in feline CKD. The histopathologic assessment of percent global glomerulosclerosis, inflammatory infiltrate, and fibrosis was performed. Senescence and nitrosative stress were evaluated utilizing p16 and iNOS immunohistochemistry, respectively. Renal telomere length was evaluated using telomere fluorescent in situ hybridization combined with immunohistochemistry. CKD cats were found to have significantly increased p16 staining in both the renal cortex and corticomedullary junction compared to adult and senior cats. Senior cats had significantly increased p16 staining in the corticomedullary junction compared to adult cats. p16 staining in both the renal cortex and corticomedullary junction were found to be significantly correlated with percent global glomerulosclerosis, cortical inflammatory infiltrate, and fibrosis scores. p16 staining also correlated with age in non-CKD cats. Average telomere length was significantly decreased in CKD cats compared to adult and senior cats. CKD cats had significantly increased iNOS staining compared to adult cats. Our results demonstrate increased renal senescence, telomere shortening, and nitrosative stress in feline CKD, identifying these patients as potential candidates for senolytic therapy with translational potential.

## 1. Introduction

Chronic kidney disease (CKD) is a common naturally-occurring disease in cats that increases in prevalence with age [1,2]. The most frequent morphologic diagnosis in cats with CKD is tubular degeneration and atrophy with interstitial inflammation, fibrosis, and glomerulosclerosis, which increase in severity with the progression of disease stages [3]. The cause of CKD in cats is unknown, but it is likely the consequence of a variety of etiologies that induce damage and lead to a final common pathway of irreversible, progressive kidney dysfunction [4,5]. Currently, there is no definitive cure for feline CKD other than renal transplant. Therefore, studies to better understand its pathophysiology and identify prospective target for therapeutic intervention are needed. Furthermore, insights into this heterogeneous population of patients with naturally-occurring disease have translational potential for aging human kidney disease patients due to similarities in histopathologic lesions despite some differences in clinical characteristics [6,7,8,9].

Consistent with the well-established paradigm of cellular senescence in aging, senescent cells accumulate in the kidney with advancing age and disease, which negatively impacts the kidney environment, including the failure of healthy regeneration and promotion of the development of fibrosis through the secretion of pro-fibrotic and pro-inflammatory mediators [10,11]. Therefore, treatment with senolytic drugs that selectively target and clear senescent cells may be an attractive therapeutic strategy for CKD [10,12]. The selective clearance of senescent cells in rodent models of renal aging and injury has resulted in improved renal function, and this modality shows potential promise as a therapeutic target in CKD [10,11,13]. Senescence likely plays a contributing role in the pathophysiology of feline CKD, given the increase in prevalence of the disease with age and the increase in senescence-associated β-galactosidase (SABG) staining previously demonstrated in cats with CKD [14]. The cell cycle inhibitor p16^Ink4a^ (p16) is a useful biomarker of senescence, and the increased expression of p16 has been documented with renal aging and disease in other species [10,15,16], though it has not been evaluated in cats.

The major drivers of senescence include critical telomere shortening, as well as factors such as injury, oxidative stress, and nitrosative stress [10,12]. Telomeres are protective structures at the ends of chromosomes that shorten with cell division and a variety of stresses until reaching a critically shortened length, at which point a state of permanent cell cycle arrest known as replicative senescence is entered. Thus, the maintenance of telomere length has important implications for aging and age-related diseases. Telomere shortening may be a consequence rather than a cause of feline CKD, as significant telomere shortening has been demonstrated in cats with CKD but not in senior cats [14]. However only a small number of senior cats have been evaluated, so further research is needed. Inducible nitric oxide synthase (iNOS) is present in the kidney under normal conditions, and it is upregulated in response to inflammation. Although normally protective, the upregulation of iNOS and the subsequent overproduction of nitric oxide (nitrosative stress) is maladaptive. Nitrosative stress has been associated with cytotoxic effects, the progression of vascular dysfunction, oxidative stress, renin–angiotensin aldosterone system (RAAS) activation, and telomere shortening [17,18,19,20,21]. The aim of the current study was to evaluate renal senescence (p16), telomere length, and nitrosative stress (iNOS) in kidney tissues from cats with naturally-occurring chronic kidney disease (CKD) and adult and senior cats without CKD to further explore the interplay between these factors in the feline disease process.

## 2. Materials and Methods

### 2.1. Case Selection

Patient databases at Colorado State University, The Ohio State University, North Carolina State University, and Royal Veterinary College, London were searched for feline necropsy cases for inclusion. Based on information obtained from the medical record, inclusion criteria for the CKD group were a serum creatinine concentration of >1.6 mg/dL (reference range: 0.8–2.4 mg/dL), urine specific gravity (USG) of <1.035, and a clinical designation of CKD based on a previous history of CKD diagnosis, physical examination findings, persistent renal azotemia, and ultrasonographic changes consistent with CKD. Cats with CKD were staged based on International Renal Interest Society (IRIS) guidelines using the entirety of historical creatinine and clinical data available for the case, and they had to have urine protein creatinine ratio (UPC) and clinical assessment of blood pressure data (non-hypertensive: documentation of blood pressure <150 mmHg; hypertensive: sub-staged as such by attending clinician, on amlodipine, or documented evidence of target organ damage) available in the medical record for the sub-staging of hypertension and proteinuria, as per IRIS guidelines [22]. Clinicopathologic data had to be available from within 2 months of euthanasia. Exclusion criteria for the CKD group included diabetes mellitus, hyperthyroidism, renal neoplasia, polycystic kidney disease, pyelonephritis, ureteral obstruction, feline infectious peritonitis, feline leukemia virus-positive, feline immunodeficiency virus-positive, non-steroidal anti-inflammatory drug administration, and chemotherapy.

Inclusion criteria for cats without CKD included serum creatinine concentration <1.6 mg/dL and USG > 1.035. Cats without CKD were defined as adult (<10 years) or senior (10 years and older). The exclusion criteria for cats without CKD included diabetes, hyperthyroidism, feline infectious peritonitis, virus-positive feline leukemia, feline immunodeficiency virus-positive, renal neoplasia (i.e., diseases that could potentially affect the kidney), NSAID administration, chemotherapy administration (i.e., drugs that could affect the kidney), urinary tract infection, hematuria, and proteinuria based on urinalysis (i.e., abnormalities of the urinary system).

A total of 54 cats with CKD (14: IRIS Stage 2; 17: IRIS Stage 3; 23: IRIS Stage 4), 19 adult controls, and 23 senior controls were available for analysis. However, the analyses were sequentially conducted over time and not all cats had materials available for subsequent analyses. A summary of signalment and serum creatinine for each group in the three analyses (p16, telomere, and iNOS) is presented in Table 1.

p16 staining was assessed in kidney tissue from 30 cats with CKD (10: IRIS Stage 2; 8: IRIS Stage 3; 12: IRIS Stage 4), 14 adult controls, and 20 senior controls. Hypertension was diagnosed in 12/30 CKD cats. Proteinuria was diagnosed in 11/30 CKD cats, borderline proteinuria was diagnosed in 13/30 CKD cats, and 6/30 CKD cats were non-proteinuric.

Telomere length was assessed in kidney tissue from 54 cats with CKD (14: IRIS Stage 2; 17: IRIS Stage 3; 23: IRIS Stage 4), 19 adult controls, and 23 senior controls. Hypertension was diagnosed in 23/54 CKD cats. Proteinuria was diagnosed in 20/52 CKD cats, borderline proteinuria was diagnosed in 17/52 CKD cats, and 15/52 CKD cats were non-proteinuric. No UPC data were available for two cats.

iNOS staining was assessed in kidney tissue from 43 CKD cats (12: IRIS Stage 2; 15: IRIS Stage 3; 16: IRIS Stage 4), 15 adult controls, and 19 senior controls. Hypertension was diagnosed in 20/43 CKD cats. Proteinuria was diagnosed in 15/43 CKD cats, borderline proteinuria was diagnosed in 15/43 CKD cats, and 11/43 CKD cats were non-proteinuric. No UPC data were available for two cats.

### 2.2. Histologic Scoring

Formalin-fixed paraffin-embedded kidney tissues were sectioned at 3 µm and stained with routine hematoxylin and eosin (H&E) and Masson’s trichrome stain. Masson’s trichrome stain was utilized/employed to assess glomerulosclerosis and interstitial fibrosis. Histologic scoring was performed by a pathologist blinded to clinical data (RC). Fifty randomly selected glomeruli per case were examined. If 2 sections of kidney were available for evaluation, then 25 glomeruli per section were examined. The number of globally sclerotic glomeruli characterized by hypo-to-acellular meshworks of collagen were counted, and the overall global glomerulosclerosis percentage was calculated. To assess fibrosis and inflammation, 10 randomly selected fields (40×) from both the cortex and corticomedullary junction were examined. Each field was assigned a score for the severity of the interstitial fibrosis (using Masson’s trichrome) and a score for inflammation (using H&E) as follows: 0 = not present; 1 = lesion present without disruption of the tubular architecture; 2 = lesion widely separates tubules; and 3 = lesion replaces tubules (scoring system used for both fibrosis and inflammation). The median score for the 10 fields was calculated for both fibrosis and inflammation in both the cortex and corticomedullary junction regions of the kidney.

### 2.3. Immunohistochemistry

Double labelling immunohistochemistry for p16/aquaporin 1 (AQP1) and iNOS/AQP1 was performed using the antibodies described in Table 2.

Three micrometer thick kidney sections from paraffin blocks were cut and mounted on positively charged slides. The sections were deparaffinized and then rehydrated with a graded ethanol series. Heat-induced epitope retrieval was performed using a pressure cooker with citrate buffer (pH 6.0) at 97 °C (20 min), followed by endogenous enzyme blocking with DEEB. Slides were processed with the sequential application of primary antibodies using an autostainer (Dako Autostainer Link 48, Agilent, Santa Clara, CA, USA) and the Envision G/2 Doublestain System Rabbit/Mouse (DAB+/Permanent Red) (Agilent, Santa Clara, CA, USA) according to manufacturer’s instructions. The slides were then counterstained with Mayer’s hematoxylin QS (Vector Laboratories; Burlingame, CA, USA; Cat #H-138 3404), dehydrated, and mounted in xylene-based mounting medium.

### 2.4. Assessment of p16 and iNOS IHC

Ten consecutive high power fields (40×) were evaluated from both cortical and corticomedullary junction regions by a pathologist blinded to clinical data (SM). Within each field, all discernable tubules were designated either AQP1+ or AQP1−, the number of p16-positive or iNOS-positive tubules was counted within each category, and an average percent positive was determined for each specimen. For p16, the following assessments were also performed. First, the semi-quantification of p16 tubular staining was performed by assigning a score to the distribution of staining (none, focal, segmental, or diffuse). This designation was made based on the predominant distribution within the effected tubules in the field. Second, the distribution of cellular p16 staining was designated as cytoplasmic, nuclear, or both if staining intensity precluded the determination of which compartment was stained. For iNOS, the intensity of tubular iNOS staining was evaluated by assigning a numeric score (0 = negative; 1 = mild; 2 = moderate; 3 = heavy) to each field.

### 2.5. Telomere FISH Slide Preparation

Formalin-fixed paraffin-embedded kidney tissues (54 cats with CKD, 19 adult controls, and 23 senior controls) were sectioned at a thickness of 3 µm. Slides were incubated at 65 °C (10 min), followed by three xylene washes (10 min). Slides were dehydrated through a graded ethanol series and rinsed in phosphate-buffered saline (PBS), then immersed in 3% hydrogen peroxide (5 min), washed in PBS, immersed in methanol (5 min) and washed in PBS. Prior to antigen retrieval, slides were immersed in 3% paraformaldehyde (10 min). Antigen retrieval was performed via immersion in a citrate buffer (Dako, Carpinteria, CA) and steaming at 125 °C (1 min) in a pressure cooker. Slides were rinsed with deionized (DI) water, dehydrated through a graded ethanol series, and stored at 4 °C.

At the time of analysis, slides were rehydrated in ethanol series (90%, 80%, 70%, and PBS), then immersed in 1% Tween-20 detergent (60 s; Sigma Aldrich, St. Louis, MO, USA), and rinsed briefly in DI water. Proteinase K (100 µL of 100 mcg/mL; Roche, San Francisco, CA, USA) was applied to slides, which were cover-slipped and incubated at 37 °C (15 min). Slides were washed in PBS (2 min), dehydrated through a graded ethanol series, and then air-dried. A Cy3-labeled peptide nucleic acid (PNA) telomere probe (TTAGGG_3_, Biosynthesis, Lewisville, TX, USA) was prepared by diluting 5 µL of probe in 36 µL of formamide (Sigma Aldrich, St. Louis, MO, USA), 12 µL of a 0.05 M TRIS buffer, 2.5 µL of 0.1 M KCl (Sigma Aldrich, St. Louis, MO, USA), and 0.6 µL of 0.1 M MgCl (Sigma Aldrich, St. Louis, MO, USA) for a final concentration of 300 ng/mL. Fifty microliters of probe mix were applied to each slide, which were then cover-slipped and denatured at 85 °C (5 min). Slides were incubated at 37 °C for 2 h, then washed in a series of 43.5 °C washes for 2.5 min each; washes 1 and 2: 50% formamide in 2X sodium citrate (SSC); washes 3 and 4: 2X SSC; and washes 5 and 6: 2X SSC and 0.1% NP40.

Following telomere probe hybridization, slides were sequentially incubated (2 h at room temperature) with two primary antibodies (concentration of 1:300): aquaporin-1 (AQP1) (mouse anti-Aquaporin 1, Millipore, Billerica, MA, USA) to identify proximal tubule segments and cytokeratin AE1/AE3 (rabbit anti-cytokeratin, Cell Marque, Rocklin, CA, USA) to identify distal tubule segments [25,26]. Anti-mouse-Alexa 488 and anti-rabbit-Alexa 647 secondary antibodies (Invitrogen, Carlsbad, CA, USA) were applied (concentration of 1:750; 30 min at room temperature) to visualize AQP1 (green) and cytokeratin (red); slides were washed as above at 43.5 °C.

Slides were counter-stained with 50 µL of DAPI (in Prolong Gold Antifade, Invitrogen, Carlsbad, CA, USA), cover-slipped, and then stored at −20 °C. Slides were processed in sets of 5–6 slides that included samples from each of the three groups.

### 2.6. FISH Image Capture, Processing and Analysis

Image Z stacks were acquired using a Nikon Eclipse 600 microscope outfitted with a Coolsnap ES camera and running Metamorph software (Molecular Devices, Sunnyvale, CA, USA). Between 15 and 20 composite images were created from 26 individual stacks (0.2 um per plane) in two different wavelengths (Dapi and Cy3). 3D deconvolution was performed using Image J software under 3D blind parameters (available as a download from http://rsb.info.nih.gov/ij/ (accessed on 5 December 2021)). In addition, the PSF (point spread function) was calculated for each picture to obtain a maximum projection of the stacks, thus allowing for the visualization and analysis of telomere signals throughout the entirety each cell nuclei. The analysis of telomere fluorescence intensity (TFI) was performed using TELOMETER, available as a download from http://demarzolab.pathology.jhmi.edu/telometer/downloads/index.html (accessed on 5 December 2021). Telomere signals from 60 nuclei per sample were analyzed using custom program settings (minimum object size: 1; maximum object size: 350; despeckle ratio: 0.3; rolling ball size: 1) and mean sample TFI was calculated. For telomere length analysis, data were summarized as the average TFI value per cat and the proportion of short telomeres per cat. Short was defined as TFI less than 100.

### 2.7. Statistical Analysis

p16 staining, histopathologic parameters, and clinicopathologic parameters were compared between groups (adult, senior, and CKD) using Kruskal–Wallis with Dunn’s post hoc analysis. The correlation between p16 staining and histologic parameters, age, and UPC was assessed with Spearman Rank. All analyses were performed using Prism (Version 7, Graph Pad Software Inc., La Jolla, CA, USA). For all analyses, *p* ≤ 0.05 was considered significant. Percent iNOS-positive and iNOS intensity scores for AQP1+ or AQP1− tubules in both the cortical and corticomedullary regions were compared between cohorts of cats using Kruskal–Wallis with Dunn’s post hoc analysis. For telomere analysis, when average TFI was the response, Proc Mixed was used to fit a mixed model including a random batch effect to compensate for any variability between batches of slides analyzed (SAS 9.4, SAS Institute Inc., Cary, NC, USA). When the proportion of short telomeres needed to be known, Proc Glimmix was used to fit beta regression with a random batch effect. Tukey adjustment was used when comparing 3 or more groups. When sub-analyses were performed within the CKD cat group, IRIS stage (2, 3, or 4), hypertension (yes or no), and proteinuria designation (no, borderline, or proteinuric) were analyzed as dichotomous variables and serum creatinine concentration and UPC were analyzed as continuous variables.

## 3. Results

### 3.1. Histopathology

Histopathology was assessed in all cats in the three analyses (Table 3, Table 4 and Table 5). Percent global glomerulosclerosis and cortical and corticomedullary inflammatory infiltrate and fibrosis scores were significantly higher in CKD cats than in adult and senior cats. No significant difference was seen in any of the histologic score categories between adult and senior cats.

### 3.2. Cellular Senescence Is Associated with Age and Increased in CKD and Senior Cat Kidneys

**Figure 1:**
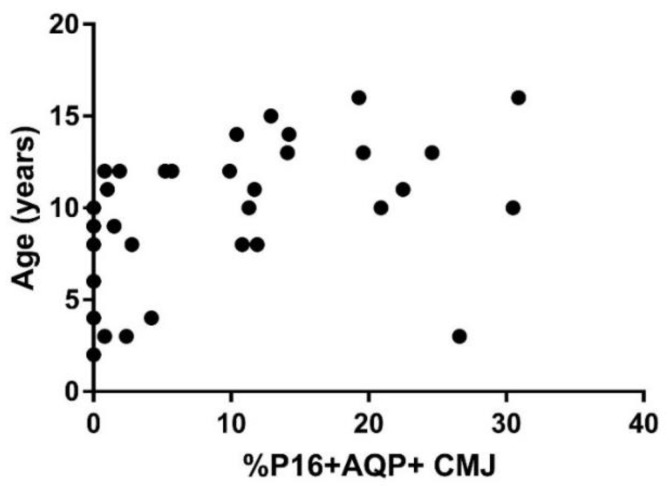
p16 staining in kidney of cats without CKD. The staining of the senescence marker p16^Ink4a^ in the corticomedullary region of the kidney was correlated with age in cats without CKD (*n* = 34; *p* = 0.002; r = 0.52). The correlation between p16 staining and age was assessed with Spearman Rank.

When the relationship between p16 staining and age was assessed in all cats without CKD, there was a significant correlation between p16 staining and age in the AQP1+ cell populations in both the renal cortex (*p* = 0.0006; r = 0.56) and corticomedullary junction (*p* = 0.002; r = 0.52; Figure 1), as well as in the AQP1− cell population in the renal cortex (*p* = 0.008; r = 0.45). No significant correlation was seen between p16 staining and age in the AQP1− cell population in the corticomedullary junction.

**Figure 2:**
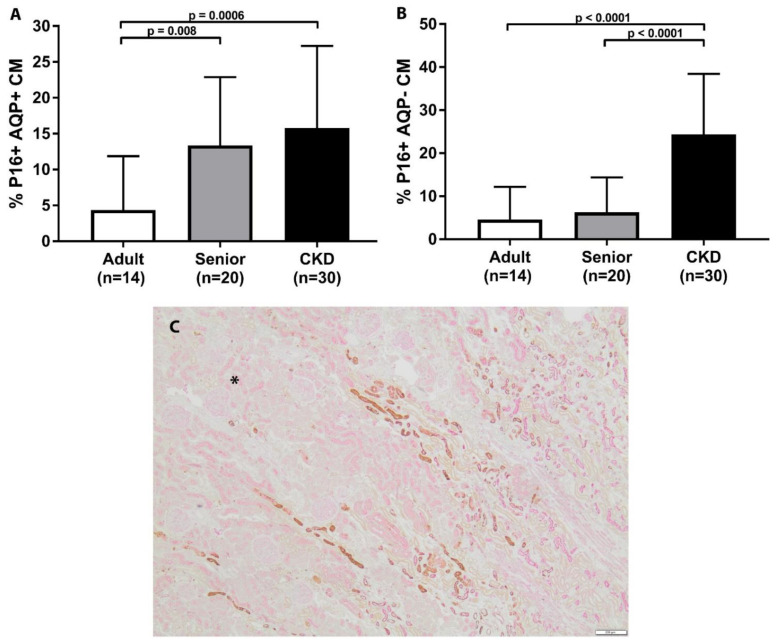
p16 staining in corticomedullary junction of feline kidney. (**A**) Senior cats had significantly increased p16 staining in AQP1+ cell populations in the corticomedullary junction in comparison to adult cats. CKD cats had significantly increased p16 staining in AQP1+ cell populations in the corticomedullary junction in comparison to adult and senior cats. (**B**) CKD cats had significantly increased p16 staining in both AQP1− cell populations in the corticomedullary junction in comparison to adult and senior cats. (mean/SD). (**C**) Double staining immunohistochemistry p16 (brown)/AQP1 (pink) in feline kidney (4×; scale bar represents 200 μm). Example of corticomedullary band of increased p16 staining in senior cat. (for orientation, cortex is in upper left corner and designated by asterisk).

A summary of p16 staining for each group is presented in Table 3. The distribution of p16 staining in all cats was either cytoplasmic or both cytoplasmic and nuclear. Senior cats had the significantly increased p16 staining of AQP1+ cell populations in the renal corticomedullary junction in comparison to adult cats (Table 3 and Figure 2). Senior cats had no significant difference in p16 staining in either the AQP1+ or AQP1− cell populations in the renal cortex when compared to adult cats. Senior cats had no significant difference in p16 staining in the AQP1− cell populations in the corticomedullary junction when compared to adult cats.

**Figure 3:**
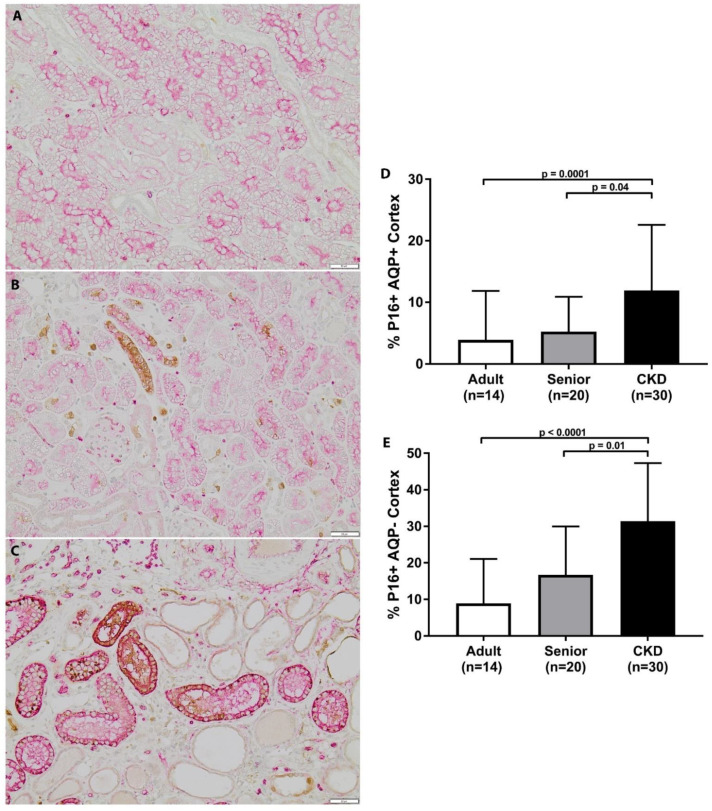
p16 staining in cortex of feline kidney. (**A**–**C**) Double staining immunohistochemistry p16 (brown)/AQP1 (pink) in feline kidney: adult (top), senior (middle), CKD (bottom) (10×; scale bar represents 50 μm). A loss of proximal tubules was apparent in CKD cats. (**D**) CKD cats had significantly increased p16 staining in AQP1+ cell populations in the renal cortex in comparison to adult and senior cats. (**E**) CKD cats had significantly increased p16 staining in AQP1− cell populations in the renal cortex in comparison to adult and senior cats (mean/SD).

CKD cats had significantly increased p16 staining in both the AQP1+ and AQP1− cell populations in the renal cortex in comparison to adult cats (Figure 3). Similarly, CKD cats had significantly increased p16 staining in both the AQP1+ and AQP1− cell populations in the renal cortex in comparison to senior cats. CKD cats had significantly increased p16 staining in both the AQP1+ and AQP1− cell populations in the renal corticomedullary junction in comparison to adult cats (Figure 2). CKD cats only had significantly increased p16 staining in the AQP1− cell populations in the renal corticomedullary junction in comparison to senior cats. Within the CKD group, p16 staining was not significantly different in CKD cats with later stage disease, hypertension, or proteinuria. p16 staining was also not significantly correlated to age, serum creatinine concentration, or UPC within the CKD cat group.

### 3.3. Telomeres Are Significantly Shortened in Kidneys of CKD Cats

**Figure 4:**
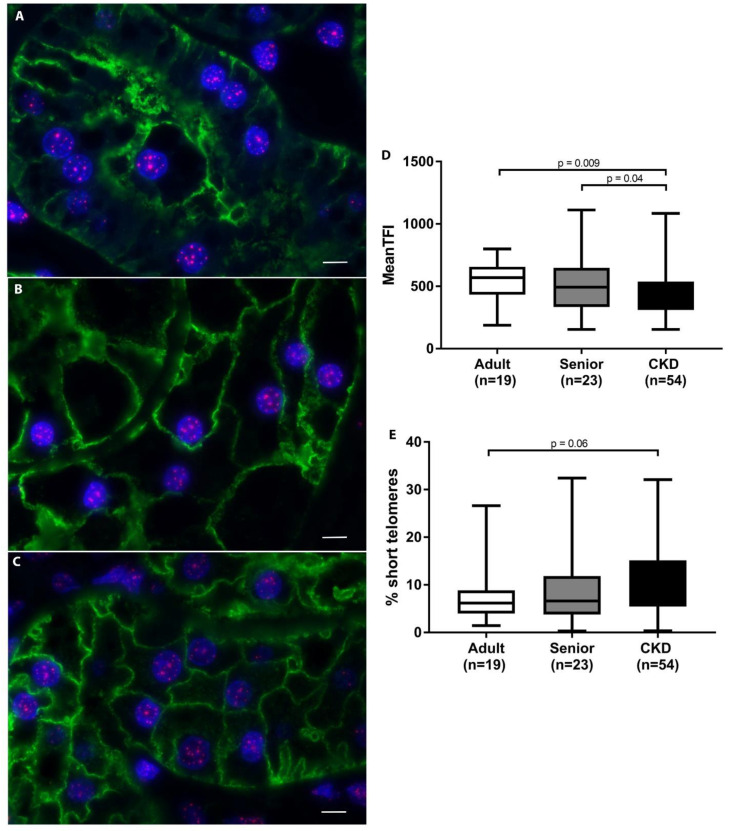
Telomere length analysis of feline kidneys. (**A**–**C**) Telomere fluorescent in situ hybridization combined with immunohistochemistry; telomeres (pink)/AQP1 (green)/nuclei (blue) proximal tubular epithelial cells in feline kidney: adult (top), senior (middle), and CKD (bottom) (100×; scale bar represents 5 µm). A loss of proximal tubules was apparent in CKD cats. (**D**) Average telomere length was significantly decreased in the proximal tubular epithelial cells of CKD cats compared to adult and senior cats. (**E**) The percentage of short telomeres in proximal tubular epithelial cells tended to be higher in CKD cats compared to adult cats. When average TFI was the response, Proc Mixed was used to fit a mixed model including a random batch effect. When the proportion of short needed to be known, Proc Glimmix was used to fit beta regression with a random batch effect. Tukey adjustment was used when comparing 3 or more groups.

A summary of telomere results for each group is presented in Table 4. The statistical analysis of mean TFI revealed no significant differences in telomere length or in the percentage of short telomeres with age in healthy cats. However, telomeres in AQP1+ proximal tubular epithelial cells were significantly shortened in CKD cats compared to both adult (*p* = 0.009) and senior cats (*p* = 0.04) (Figure 4). Percent short telomeres tended to be higher in CKD cats compared to adult cats (*p* = 0.06). Interestingly, CKD cats with hypertension displayed significantly shorter telomeres (*p* = 0.01), as well as a trend towards higher percentages of short telomeres (*p* = 0.06), compared to CKD cats without hypertension. Within the CKD cat group, there were no significant differences in mean telomere length or percent short telomeres with IRIS stage or proteinuria designation (Table 5). Additionally, neither mean telomere length nor percent short telomeres were correlated with serum creatinine concentration or UPC within the CKD group.

### 3.4. Nitrosative Stress Is Increased in Feline CKD

**Figure 5:**
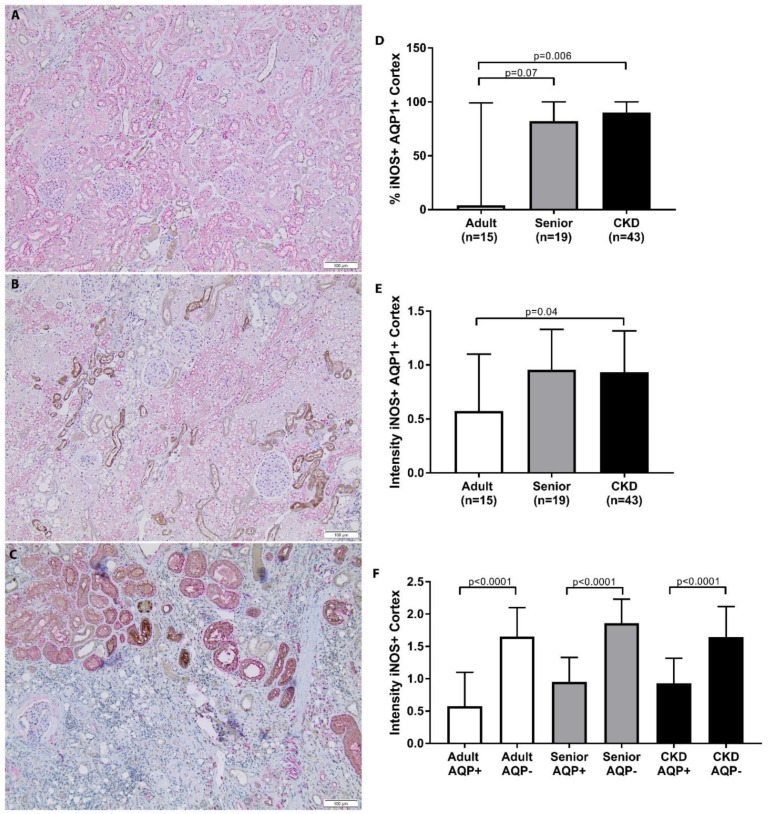
iNOS staining in cortex of feline kidney. (**A**–**C**) Double staining immunohistochemistry p16 (brown)/AQP1 (pink) in feline kidney: adult (top), senior (middle), and CKD (bottom) (10×; scale bar represents 50 µm). A loss of proximal tubules was apparent in CKD cats. (**D**) CKD cats had significantly increased iNOS staining in AQP1+ cell populations in the renal cortex in comparison to adult cats. Senior cats trended towards significantly increased iNOS staining in AQP1+ cell populations in the renal cortex in comparison to adult cats; median/range. (**E**) CKD cats had a significant increase in the intensity of iNOS staining in the renal cortex in comparison to adult cats; mean/SD. (**F**) AQP1− tubules had an increased intensity of iNOS staining in comparison to AQP1+ tubules in the cortex and corticomedullary junction of all groups. iNOS staining was compared between groups using Kruskal–Wallis test with Dunn’s post hoc analysis.

A summary of the iNOS staining scoring for each group is presented in Table 6. CKD cats had significantly increased iNOS staining in the AQP1+ cell populations in the renal cortex in comparison to adult cats (Figure 5). Senior cats trended towards significantly increased iNOS staining in the AQP1+ cell populations in the renal cortex in comparison to adult cats. There was no significant difference between groups in iNOS staining in the AQP1+ cell populations in the corticomedullary junction or in the AQP1− cell populations in the cortex or corticomedullary region. Percentage iNOS staining was not significantly different between CKD cats with or without hypertension, with or without proteinuria, or between different IRIS stages. When the intensity of iNOS staining was assessed, there was a significant difference in the intensity of iNOS staining in the cortex of CKD cats in comparison to adult cats (Figure 5). AQP1− tubules presented an increased intensity of iNOS staining in comparison to AQP1+ tubules in the cortex and corticomedullary junction of all groups (Figure 5).

### 3.5. Glomerulosclerosis, Inflammation, and Fibrosis Is Correlated with p16 Staining but Not Telomere Length or iNOS

p16 staining in both the AQP1+ and AQP1− cell populations in the renal cortex was significantly correlated with percent global glomerulosclerosis and cortical inflammatory infiltrate and fibrosis scores (Table 7). p16 staining in the AQP1+ cell populations in the corticomedullary junction was not significantly correlated with corticomedullary inflammatory infiltrate scores, but it was significantly correlated with corticomedullary fibrosis scores (Table 7). p16 staining in the AQP1− cell populations in the corticomedullary junction was significantly correlated with both corticomedullary inflammatory infiltrate and fibrosis scores (Table 7). There was no significant correlation between telomere length or percentage short telomeres and glomerulosclerosis, cortical inflammation, or fibrosis. There was no significant correlation between iNOS staining and glomerulosclerosis, cortical inflammation, or fibrosis.

### 3.6. Telomere Length Is Not Correlated with p16 or iNOS Staining

When correlation analyses were performed in the subset of samples for which all three analyses were conducted (20 CKD, 9 adult, and 16 NG), there was no correlation between telomere length and either p16 AQP1+ cortex or iNOS AQP1+ cortex. There was also no correlation between percent short telomeres and either p16 AQP1+ cortex or iNOS AQP1+ cortex

## 4. Discussion

We speculated that senescence plays a key role in the etiology of feline CKD given the increase in the prevalence of disease with age and the increased expression of senescence-associated β-galactosidase [14]. Our results suggest that p16 is a mediator of renal senescence in both senior cats and cats with CKD, as cats with CKD exhibited accelerated p16-mediated senescence in both the cortex and corticomedullary junction of the kidney, beyond what would be expected for normal aging. Furthermore, p16 staining was positively correlated with glomerulosclerosis, interstitial inflammation, and fibrosis in the kidney.

p16 staining was not associated with the severity of disease, as determined by serum creatinine concentration or IRIS CKD stage. Serum creatinine concentration may be lower in patients with muscle wasting and therefore may not be a reliable indicator of the severity of disease in some cases. Given the retrospective nature of the current study, it was not possible to obtain complete information on the muscle mass of the cats. Symmetric dimethylarginine (SDMA) is a biomarker of glomerular filtration rate that is not effected by muscle mass like serum creatinine [27]; however, data for this biomarker were not available for the cats in the study. Given the limitations of serum creatinine, the correlation with histopathologic scoring is a more robust finding and is similar to that described in human CKD patients, in which tubular p16 is correlated with tubular atrophy, interstitial fibrosis, and glomerulosclerosis [15,28,29].

Senior cats were observed to have increased p16 staining in the corticomedullary region of the kidney compared to young cats, while staining in the cortex was not significantly increased. The location of senescent cells appears to depend on the etiology of the instigating stressor and the location of initial injury [12,15,30]. For example, during renal aging in humans, senescence is predominantly found in the proximal tubular cells of the renal cortex (versus the medulla), likely due to increased oxidative and cellular stress in this cellular population [12,15]. In cats, recent work using kidney injury molecule-1 as a biomarker for acute kidney injury has demonstrated that there is a segment of proximal tubule in the corticomedullary junction that is particularly sensitive to ischemic injury [31]. Therefore, it is plausible that increased p16 staining in this area represents evidence of prior damage events associated with the increased susceptibility of this nephron segment in this species. This would be consistent with current theories regarding the etiology of feline CKD; namely that serial subclinical multifactorial injury events culminate in clinical disease later in life and injuries are further exacerbated by the susceptibility of the aging kidney [4,5,32].

Consistent with our previous findings, replicative senescence was associated with telomere shortening in CKD cats in comparison to normal controls [14]. Several processes likely contribute to telomere attrition in CKD, such as oxidative stress, renal ischemia, and hypertension, all of which have been associated with shortened renal telomeres [33,34,35]. In CKD, the renal environment is subjected to chronic inflammation, oxidative stress, and hypoxia—factors known to negatively influence telomere length [3,19,21,36,37]. Specifically in cats, normal tubules contain large amounts of lipid, and when damage occurs, lipid is released into the interstitium, thus becoming a nidus for inflammation [3]. When considered together with similar observations in human CKD patients [38,39,40,41,42], it is not surprising that telomeres are shortened in cats with CKD, a scenario that would eventually result in critically short telomeres and the triggering of replicative senescence [36,37,43,44,45].

Oxidative stress is of particular relevance to the induction of replicative senescence because telomeres are exceptionally susceptible to oxidative injury, and replication combined with an impaired ability to repair oxidative damage causes rapid telomere shortening and dysfunction [46,47]. Oxidative stress is also a major driver of p16-mediated senescence [48]. Tubular epithelial cells have high metabolic activity and thus a high production rate of reactive oxygen species (ROS), which is further exacerbated when tubule loss leads to the hyperfiltration of remaining nephrons [5]. The generation of ROS is also promoted by hypoxia associated with fibrosis and capillary rarefaction, RAAS activation, anemia, hyperphosphatemia, and uremic toxins [5]. Evidence of oxidative stress has been demonstrated in feline CKD [49,50,51,52]. Cats with CKD display the activation of antioxidant defense mechanisms and lower antioxidant capacity than normal controls, consistent with imbalance between oxidative stress and antioxidant defense mechanisms [49]. Additionally, oxidative stress, as measured by increased urinary F_2_-isoprostanes, may be important even in early stages of disease [50]. Oxidative stress also has been therapeutically targeted with dietary supplements of vitamin E, C, and beta-carotene that resulted in reduced oxidative stress in CKD cats, as measured by serum concentrations of 8-hydroxy-2′-deoxyguanosine [51]. Therefore, it is plausible that oxidative stress contributes to p16-mediated senescence, as well as to dysfunctional telomere-mediated replicative senescence observed in cats with CKD.

The assessment of oxidative stress utilizing 8-hydroxy-2′-deoxyguanosine immunohistochemistry was attempted in the current study, but significant non-specific staining in the feline kidney impeded interpretation. As an alternative approach, nitrosative stress was assessed, as this had not previously been studied in feline CKD. Nitrosative stress, particularly in combination with oxidative stress, contributes to the imbalance between ROS and antioxidants and has a direct damaging effect on telomeres, thus acting as a driver of senescence, vascular dysfunction, epithelial mesenchymal transition, and development of fibrosis [17,18,19,20,21]. Despite the basal staining of iNOS in the feline kidney, particularly in the distal tubules, iNOS staining was increased in the proximal tubules of the renal cortex of cats with CKD in comparison to adult cats. Increased iNOS staining in feline CKD provides additional mechanistic insight into the telomere shortening observed in the kidneys of cats with CKD, as well as in the development of renal senescence in general. Although not commonly considered as a therapeutic target in feline medicine, additional investigation into mitigating nitrosative stress is warranted.

In humans, telomeres in the renal cortex shorten in an age-dependent manner [53,54], and telomere attrition has been described as a causal factor for CKD [42]. In contrast, we found no evidence of telomere shortening in the kidneys of senior cats without CKD, despite evaluating a large number of samples here and elsewhere [14]. Recent evidence suggests that telomere dysfunction can occur independent of length and may contribute to senescence [47]. In one study, increased p16 was reported in the lung tissue of COPD patients, but damaged telomeres (those associated with DNA damage response proteins) were not significantly shorter than undamaged telomeres [55]. Thus, the relationship between p16 expression and telomere length is unclear. It has been postulated that telomeres sense stresses and maintain stability by limiting the replication of cells that have significant genomic damage [47]. Therefore, given significantly increased p16 and the trend towards increased iNOS expression in senior cats, the additional study of oxidative stress and DNA damage responses is merited in this patient population to further explore the transition from renal aging to disease.

Hypertension is a common comorbidity in feline CKD, with 20–65% of patients typically effected [56,57,58,59]. Interestingly, a significant decrease in telomere length was observed in hypertensive CKD cats in our study. These findings are similar to several other studies in humans and rodents, where hypertension has been associated with short telomeres, both within the kidney and in leukocytes [35,60,61]. Importantly, in BPH/2J hypertensive mice, renal telomere length shortens after the development of hypertension [59], a finding that implies hypertension in feline CKD may contribute to telomere shortening beyond that caused by the disease itself. Significantly shorter telomeres were seen in hypertensive cats despite antihypertensive therapy, which is contrary to expectation based on a study in humans in which antihypertensive therapy was associated with the amelioration of telomere attrition [60]. However, in that study, patients were followed over time and exact data on blood pressure measurements were available. As our study utilized tissues from necropsy archives, the ability to assess specific values of blood pressure data was limited, and therefore only a determination of “yes” or “no” for the presence of hypertension could be made. Additionally, blood pressure data were not available for non-CKD patients because primary hypertension was perceived to be relatively uncommon in cats without CKD at the time the cats were alive. Since that time, newer data have demonstrated that the prevalence of hypertension in the healthy elderly cat population is approximately 8–12% [61,62,63]. No relationship between p16 or iNOS staining and a diagnosis of hypertension was found. This was in contrast to a study in human IgA nephropathy patients demonstrating that the degree of hypertension was correlated with p16 staining; however, assessing hypertension as a continuous variable may not yield the same conclusion as when assessed as a dichotomous variable as it was in our study [29].

Proteinuria was not found to be associated with senescence, telomere length, or nitrosative stress. These findings are perhaps not surprising because feline CKD is most commonly characterized by tubulointerstitial disease as opposed to primary glomerular disease, as is seen in dogs and humans [3,64]. Thus, in cats, proteinuria is typically secondary to tubular dysfunction and glomerulosclerosis, which increase in prevalence with disease severity [3]. Additionally, in humans with glomerular disease, the p16 staining of glomerular cell nuclei was not found to be correlated with degree of proteinuria [30], so there may not be a direct association.

Once senescence is initiated, effected cells further exacerbate the microenvironment by acquiring a senescence-associated secretory phenotype (SASP), which consists of an array of pro-fibrotic and pro-inflammatory mediators including IL-6, IL-8, MMPs, MCP-1, and TGF-β [10,11]. Paracrine messaging from senescent cells has as negative effect on neighboring cells, potentially inducing senescence, inhibiting cell renewal, and contributing to the formation of fibrosis [11]. Although SASP factors were not directly assessed in our study, there is some evidence for SASP in feline CKD, despite this area of research being hampered by a lack of optimally-performing species-specific ELISA assays [65]. Elevated concentrations of IL-8 and TGF-β have been found in the urine of cats with CKD, and urinary TGF-β has been associated with both the degree of fibrosis and inflammation on histopathology, as well as with the initial development of azotemic CKD [65,66]. In studies assessing gene transcription in feline CKD (both ischemia-induced and naturally occurring), kidneys from CKD cats have been shown to have significantly higher transcript levels of HIF1A, MMPs, and TGF-β [37,45]. VEGF is decreased in feline CKD [37,45,65], which differs from other SASP literature [11]. Nonetheless, there are data to support the induction of senescence and the further exacerbation of disease via the development of SASP in feline CKD.

Limitations of this study include its retrospective nature, which could have influenced the reliability of the clinical data. Although all available medical records and historical clinicopathologic data for each case were utilized in determining IRIS stage and sub-stage, clinicopathologic data were sometimes not available near the time of death, so a cut-off of 2 months was used for inclusion in the study. Additionally, the inability to perform all techniques (telomere, p16, and iNOS analysis) on all kidneys led to a limited number of cats for some subgroup analyses (e.g., influence of hypertension and proteinuria).

## 5. Conclusions

Taken together, the results of this study support p16 as a mediator of renal senescence in senior cats and cats with CKD. Although increased p16-mediated senescence was observed with age, cats with CKD exhibited accelerated senescence, beyond that which would be expected for aging alone. Telomere shortening and nitrosative stress were also present in feline CKD and likely further contribute to renal senescence. Future work is warranted to determine if senescence predisposes senior cats to the development of CKD. Importantly, these results suggest that feline CKD may be amenable to novel and translatable senolytic therapeutic strategies.

## Figures and Tables

**Table 1:** Number of cats, signalment, and creatinine for each analysis group. Median (range).

	Adult Cats	Senior Cats	CKD Cats
**p16**	**(*n* = 14)**	**(*n* = 20)**	**(*n* = 30)**
Age (years) ^a,b^	5 (2–9)	12 (10–16)	15 (3–20)
Sex	MN (8) FS (6)	MN (13) FS (7)	MN (20) FS (10)
Creatinine (mg/dL) ^b,c^	0.8 (0.3–1.5)	1.1 (0.4–1.5)	3.9 (1.7–10)
**Telomere**	**(*n* = 19)**	**(*n* = 23)**	**(*n* = 54)**
Age (years) ^a,b^	4 (0.6–9)	12 (10–16)	15 (3–21)
Sex	MN (13) FS (6)	MN (13) FS (10)	MN (35) FS (19)
Creatinine (mg/dL) ^b,c^	1.0 (0.4–1.5)	1.1 (0.4–1.5)	4.3 (1.7–13.6)
**iNOS**	**(*n* = 15)**	**(*n* = 19)**	**(*n* = 43)**
Age (years) ^a,b^	4 (0.6–9)	12.5 (10–16)	15 (4–21.4)
Sex	MN (10) FS (5)	MN (11) FS (8)	MN (28) FS (15)
Creatinine (mg/dL) ^b,c^	1.0 (0.3–1.5)	1.1 (0.5–1.5)	3.9 (1.7–10)

CKD: chronic kidney disease; MN: male neutered; FS: female spayed. ^a^: significant difference between adult and senior; ^b^: significant difference between adult and CKD; ^c^: significant difference between senior and CKD.

**Table 2:** Antibodies used for dual immunohistochemistry.

Marker	Type, Clone, Reference	Supplier	Dilution
AQP1	Rabbit polyclonal anti-aquaporin-1 AP, AB2219 [14]	Millipore; Billerica, MA, USA	1:1000
p16	Mouse monoclonal anti-human p16, Cat#550834 [23]	BD Biosciences; San Jose, CA, USA	1:25
iNOS	Rabbit polyclonal anti-inducible nitric oxide synthase, NB300-605 [24]	Fisher Scientific, Waltham, MA, USA	1:100

**Table 3:** Histopathologic scoring and p16 staining for each group. Median (range).

	Adult Cats (*n* = 14)	Senior Cats (*n* = 20)	CKD Cats (*n* = 30)
% Global GS ^b,c^	0 (0–14)	2 (0–40)	15 (0–92)
Inflammatory Infiltrate Score Cortex ^b,c^	0 (0–1)	0 (0–0.7)	0.9 (0–2.9)
Fibrosis Score Cortex ^b,c^	0 (0–1)	0 (0–0.6)	0.8 (0–2.7)
Inflammatory Infiltrate Score CMJ ^b,c^	0 (0–1)	0 (0–0.5)	1.5 (0.2–2.5)
Fibrosis Score CMJ ^b,c^	0.1 (0–1.4)	0 (0–1)	1.5 (0.3–2.5)
% p16+AQP1+ Cortex ^b,c^	0 (0–22.3)	2.6 (0–20.6)	10.6 (0–55)
% p16+AQP1− Cortex ^b,c^	3.7 (0–35)	15.5 (0–42.6)	28.5 (5.3–65.9)
SQ p16+AQP1+ Cortex ^b,c^	0 (0–2)	0 (0–1.5)	1 (0–3)
SQ p16+AQP1− Cortex ^b,c^	0 (0–1)	0.25 (0–1.5)	1 (0–3)
% p16+AQP1+ CMJ ^a,b^	1.2 (0–26.6)	12.3 (0–30.9)	12.2 (1.2–48.2)
% p16+AQP1− CMJ ^b,c^	1.1 (0–26.5)	2.7 (0–25.8)	23.8 (1.7–52)
SQ p16+AQP1+ CMJ ^a^	0 (0–2)	1 (0–2)	0.5 (0–2.5)
SQ p16+AQP1− CMJ ^b,c^	0 (0–0.5)	0 (0–0.5)	1 (0–3)

CKD: chronic kidney disease; GS: glomerulosclerosis; CMJ: corticomedullary junction; SQ: semi-quantitative. ^a^: significant difference between adult and senior; ^b^: significant difference between adult and CKD; ^c^: significant difference between senior and CKD.

**Table 4:** Histopathologic scoring and telomere length for each group. Results are median (range) for histopathology lesions and mean ± SE for telomere length.

	Adult Cats (*n* = 19)	Senior Cats (*n* = 23)	CKD Cats (*n* = 54)
% Global GS ^b,c^	0 (0–16)	2 (0–40)	21 (0–92)
Inflammatory Infiltrate Score Cortex ^b,c^	0 (0–1.0)	0.1 (0–0.7)	1.1 (0–2.9)
Fibrosis Score Cortex ^b,c^	0 (0–1.0)	0 (0–0.8)	0.8 (0–2.7)
Mean Telomere TFI ^b,c^	556 ± 41.9	524.2 ± 39.7	429.4 ± 31.4
% Short Telomeres ^d^	7% ± 1%	8% ± 1%	10% ± 1%

CKD: chronic kidney disease; GS: glomerulosclerosis; TFI: telomere fluorescent intensity. ^b^: significant difference between adult and CKD; ^c^: significant difference between senior and CKD; ^d^: statistical trend between adult and CKD.

**Table 5:** Sub-analyses of telomere length and % short telomeres between age groups in cats without CKD, CKD IRIS stages, and sub-stages of proteinuria and hypertension (mean ± SE).

	Mean Telomere TFI	*p*-Value	% Short Telomeres	*p*-Value
**Normal Cats**		0.749		0.335
**Age (years)**				
<5 (*n* = 10)	538.4 ± 66.4		6% ± 1%	
5–9 (*n* = 9)	600.3 ± 68.5		6% ± 2%	
10–15 (*n* = 20)	542.0 ± 50.3		7% ± 1%	
>15 (*n* = 3)	489.99 ± 117.9		11% ± 4%	
**CKD IRIS Stage**		0.9313		0.9101
IRIS Stage 2 (*n* = 14)	440.9 ± 47.3		10% ± 2%	
IRIS Stage 3 (*n* = 17)	452.5 ± 46.1		10% ± 2%	
IRIS Stage 4 (*n* = 23)	429.0 ± 41.7		11% ± 2%	
**CKD Proteinuria**		0.572		0.770
NP (*n* = 15)	431.1 ± 50		11% ± 2%	
BP (*n* = 17)	418 ± 43.3		10% ± 2%	
P (*n* = 20)	470.2 ± 42.3		10% ± 2%	
**CKD Hypertension**		**0.03**		0.057
Yes (*n* = 23)	384.8 ± 38		13% ± 2%	
No (*n* = 31)	481.2 ± 34.7		9% ± 1%	

CKD: chronic kidney disease; IRIS: International Renal Interest Society; NP: non-proteinuric; BP: borderline-proteinuric; P: proteinuric.

**Table 6:** Histopathologic scoring and iNOS staining for each group. Median (range).

	Adult Cats (*n* = 15)	Senior Cats (*n* = 19)	CKD Cats (*n* = 43)
% Global GS ^b,c^	0 (0–14)	2 (0–40)	21 (0–92)
Inflammatory Infiltrate Score Cortex ^b,c^	0 (0–1)	0 (0–0.7)	1.1 (0–2.9)
Fibrosis Score Cortex ^b,c^	0 (0–1)	0 (0–0.8)	0.7 (0–1.9)
Inflammatory Infiltrate Score CMJ ^b,c^	0 (0–1)	0 (0–0)	1.3 (0–2.5)
Fibrosis Score CMJ ^b,c^	0.1 (0–1.4)	0 (0–1)	1.5 (0.2–2.1)
% iNOS+ AQP1+ Cortex ^a,b^	4 (0–99)	82 (0–100)	90 (0–100)
% iNOS+ AQP1− Cortex	64 (35–98)	84 (28–100)	92 (21–100)
INT iNOS+ AQP1+ Cortex ^b^	1 (0–1.2)	1 (0–1.8)	1 (0–2)
INT iNOS+ AQP1− Cortex	1.5 (1–2.9)	1.9 (1–2.9)	1.6 (1–2.8)
% iNOS+ AQP1+ CMJ ^d,e^	37 (0–100)	94.5 (0–100)	89 (0–100)
% iNOS+ AQP1− CMJ	50 (4–100)	91.5 (1–100)	90.5 (3–100)
INT iNOS+ AQP1+ CMJ	1 (0–1)	1 (0–2)	1 (0–2)
INT iNOS+ AQP1− CMJ	1.3 (1–2)	1.2 (1–2.1)	1 (0–2)

CKD: chronic kidney disease; GS: glomerulosclerosis; CMJ: corticomedullary junction; INT: intensity. ^a^: significant difference between adult and senior; ^b^: significant difference between adult and CKD; ^c^: significant difference between senior and CKD; ^d^: statistical trend between adult and senior; ^e^: statistical trend between adult and CKD.

**Table 7:** Correlation between p16 and staining and mean histopathologic scores.

	% GGS	Inflammatory Infiltrate Score	Fibrosis Score
**Cortex**			
% p16+AQP1+	*p* = 0.003	*p* = 0.006	*p* = 0.0008
	r = 0.37	r = 0.35	r = 0.41
% p16+AQP1−	*p* = 0.0002	*p* < 0.0001	*p* < 0.0001
	r = 0.46	r = 0.53	r = 0.53
**CMJ**			
% p16+AQP1+	NA	NS	*p* = 0.04
			r = 0.26
% p16+AQP1−	NA	*p* < 0.0001	*p* < 0.0001
		r = 0.66	r = 0.72

GGS: global glomerulosclerosis; CMJ: corticomedullary junction; NA: not applicable; NS: not significant.

## Data Availability

Data is contained within the article.
